# Quantification of the Life Time Flight Capabilities of the South American Palm Weevil, *Rhynchophorus palmarum* (L.) (Coleoptera: Curculionidae)

**DOI:** 10.3390/insects12020126

**Published:** 2021-02-01

**Authors:** Mark S. Hoddle, Christina D. Hoddle, Ivan Milosavljević

**Affiliations:** Department of Entomology, University of California, Riverside, CA 92521, USA; mark.hoddle@ucr.edu (M.S.H.); christina.hoddle@ucr.edu (C.D.H.)

**Keywords:** dispersal, flight mill, kurtosis

## Abstract

**Simple Summary:**

The South American palm weevil, *Rhynchophorus palmarum*, is an invasive pest that has killed thousands of ornamental palms in San Diego County in California, USA. Emerging management plans for this pest need to consider the flight capabilities of this insect, which are not well understood. To address this shortcoming, flight mills, a type of computerized insect “merry-go round” that measures how far weevils can fly in the laboratory, were used to quantify the flight distances of 101 weevils that were flown repeatedly over the course of their lifetimes. The results indicate that weevils are strong flyers capable of flying numerous times before dying of natural causes. Over their lifetimes, weevils, on average, covered distances that cumulatively totaled >220 km. One female weevil flew an impressive cumulative distance of approximately 806 km over the course of nine consecutive flights before dying.

**Abstract:**

The life time flight capabilities of an invasive palm pest, *Rhynchophorus palmarum*, were assessed using flight mill assays under controlled conditions in the laboratory. A total of 101 weevils were used for experiments and subjected to repeat flight assays. A total of 17 flight trials were run, of which the first 14 provided useful data prior to weevil death. Male and female weevils exhibited a strong capacity for repeat long distance flights. Flight metrics of interest were not affected by weevil sex or mating status. Cumulative lifetime flight distances for male and female *R. palmarum* averaged ~268 km and ~220 km, respectively. A maximum lifetime cumulative flight distance of ~758 km and ~806 km was recorded for one male of unknown mating status and one unmated female weevil, respectively. Dispersal data for individual flights (i.e., trials 1 through 9, 10–14 combined) and all flight trial data (i.e., flights 1–14 combined) exhibited platykurtic distributions. The results presented here may have important implications for modeling the spread of this invasive pest and for the development of monitoring and management plans.

## 1. Introduction

Quantification of the dispersal capabilities and distribution patterns of distance data can assist with understanding the factors affecting the observed patterns of spread for an invasive pest [1,2,3]. This insight may help with the development of management strategies (e.g., delineation of quarantine boundaries) for invasive pests, such as vagile insects, which are capable of spreading by flight [4]. With respect to insects, measuring dispersal by flight in the field is challenging [5]. Flight mills are a laboratory-based tool used for quantifying the distances insects are capable of flying [6,7]. Flight mill assays subject test insects to unnatural flight conditions (i.e., flight is tethered and insects move in circular patterns) in an optimized environment (i.e., no wind and temperature and humidity are controlled) in the laboratory. These operational characteristics would not be experienced by flying insects in nature, and as such, flight mill studies are recognized as being a highly artificial approach to measuring insect flight capabilities [6,7,8].

Regardless of the acknowledged shortcomings, flight mill assays are useful for investigating the effect of factors such as size, satiation and mating status, and sex, for example, on flight metrics of interest (e.g., distances flown, flight velocity, frequency and length of flight bouts) [4,9,10,11,12]. Potentially, dispersal data may provide an indication of the type of distribution to which flight distances conform. An invasive pest, for example, may have dispersal distances that have a leptokurtic distribution, which has “fat” or “heavy” tails, because a significant number of long distance events fall within curve extremities (i.e., the tails) [1,2,3,9]. Conversely, tails may be platykurtic and have “light” or “thin” tails because relatively few extreme values occur in curve tails. Therefore, these two dispersal distributions are skewed towards distances that are lesser (i.e., platykurtic) or greater (i.e., leptokurtic) than would be predicted by a mesokurtic distribution (i.e., normal distribution) [1,3,4,9,10]. Consequently, the spread of some invasive pests may be slower or faster and cover lesser or greater distances than anticipated because dispersal is non-mesokurtic [1,2].

In 2011, the South American palm weevil, *Rhynchophorus palmarum*, was detected for the first time in San Diego County, California USA. Around 2015, weevil populations established in San Ysidro, in southern San Diego County, ~5 km north of infestations that were detected in Tijuana, Baja California, Mexico in 2010 [13]. This weevil is a palm specialist (Arecaceae: Arecales) and is native to parts of Mexico, Central and South America, and the Caribbean [14]. Palm hosts include African oil palm (*Elaeis guineensis* Jacq.,) Canary Islands date palm (*Phoenix canariensis* Chaub.), coconut (*Cocos nucifera* L.), and edible date palm (*P. dactylifera* L.) [14]. Since establishment in San Diego County, more than 10,000 ornamental *P. canariensis*, a highly preferred host, have been killed by *R. palmarum* [15]. Weevil larvae kill palms by feeding in the apical meristematic region [16]. In the native range, *R. palmarum* vectors a plant pathogenic nematode, *Bursaphelenchus cocophilus* (Cobb) (Aphelenchida: Parasitaphelenchidae), which causes red ring disease, a lethal ailment afflicting infested palms [17,18].

*Rhynchophorus palmarum* was first recorded in Baja California Sur Mexico in 2000 attacking Mexican fan palm, *Washingtonia robusta* Wendl., in Todos Santos located at the southern tip of the Baja California peninsula [19], which is ~1500 km south of Tijuana. It is probable that weevils spread naturally from Todos Santos into new areas of the peninsula by flying, possibly reaching Tijuana without human assistance over a 10-year period. The assumption of possible natural dispersal by flight was supported by data generated from flight mill studies. These laboratory assays indicated that *R. palmarum* is a strong flyer with potential to disperse long distances via flight over a 24 h period and flight distance data conformed to a platykurtic distribution [4]. A notable shortcoming with the study by Hoddle et al. [4] is that assays were time- (i.e., 24 h duration) and trial-limited (i.e., 87 weevils were flown once).

Under field conditions, insects are likely to fly more than once during their life time. Despite a probable proclivity for this obvious behavior, few flight mill studies have investigated and quantified repeat flight events by the same experimental insects after being provided a recovery period (i.e., days) between flight assays to rest and feed [20]. *Rhynchophorus palmarum* is a large, long-lived insect capable of flying more than once during its life time. In contrast to the previous 24 h flight mill study by Hoddle et al. [4] with *R. palmarum*, a better understanding of flight and dispersal capabilities of this insect would be derived by quantifying, through repeat flight mill assays, the potential life time flight capabilities of *R. palmarum*.

In work reported here, repeat flight mill assays were conducted with 101 *R. palmarum* to determine, among other things, the cumulative distances that test insects are capable of flying over their lifetime and whether kurtoses of dispersal distributions of flight distances differ across repeat assays. To achieve this, weevils were flown multiple consecutive times on flight mills, with recovery periods, until death from natural causes occurred. These life time repeat flight data have significant practical applications for modeling spread of *R. palmarum* in invaded areas and predicting rates and patterns of spread into new areas, as well as for assisting with the design and implementation of control, containment, monitoring, and quarantine programs for this pest.

## 2. Materials and Methods

### 2.1. Source and Maintenance of R. palmarum Used for Flight Mill Studies

A total of 101 *R. palmarum* (i.e., 61 females and 40 males) were used for flight mill studies. A total of 67 weevils (40 females and 27 males) were of unknown age and mating status, and were captured alive in four bucket traps set at the Sweetwater Reserve, in Bonita San Diego County, California, an area with hundreds of naturalized *P. canariensis*, many of which are infested with *R. palmarum* (see Hoddle et al. [4] for field trapping details). In addition to field captured weevils, unmated females [21] and males [13] were reared from cocoons collected from three weevil infested *P. canariensis* that were removed in Chula Vista, Bonita, and San Diego, San Diego County over the same time period weevils were field captured in bucket traps (25 October 2016–10 May 2019).

Field collected weevils were moved under California Department of Food and Agriculture Permit 3289 to the Insectary and Quarantine Facility (IQF) at the University of California, Riverside and maintained in a temperature (23.26 °C ± 0.84) and humidity (% RH 39.38% ± 0.15%) controlled room with a light/dark cycle set at 14:10 (lights on at 06:00 and off at 20:00). All adult weevils were maintained individually in labeled clear ventilated plastic containers (height 18 cm, width 13 cm, depth 13 cm) that were provisioned with pieces of cut apple, longitudinally split sugar cane, and sections of banana with skin. These foods were changed every 2–3 days when containers were cleaned to remove condensation and decomposing foods. Weevils on this mixed fruit diet were given an average of 11 ± 0.20 (±SE) (range 5–16 days) rest days between flight mill trials.

### 2.2. Flight Mills and Experimental Set Up

Flight mills used in experiments were custom made from aluminum blocks at the University of California Riverside. Each flight mill was connected to a laptop computer via a USB4 Encoder Data Acquisition Device (US Digital, Vancouver, WA, USA). Custom software recorded flight data and macros developed for Microsoft Excel analyzed raw data from each individual flight mill. Summary data of interest included total distance flown, average velocity, total time spent flying, and maximum and mean flight bout times and distances (total cumulative flight bouts per weevil had to surpass 1 km in a 24 h flight assay before being included in analyses). Weevils used in experiments were attached dorsally by their thorax to an “L” shaped metal plate (~0.59 mm diameter by 28 mm long) flattened at one end for insect adhesion. A small drop of hot glue was applied to the thorax of the experimental weevil and the flattened end of the harness was submersed in the glue. Once glue dried, harnesses with attached weevils were affixed to 30.5 cm flight mill arms made of 0.5 mm carbon steel via a socket crimp (model 809–043, Glenair, Glendale CA, USA). Once attached, weevils were inspected to make sure they could open their elytra and move their legs. Modeling clay of the same approximate weight as tethered weevils was then placed on the opposite end of the flight mill arm to counterbalance the weight of the adult beetle. Lopez et al. [11] and Hoddle et al. [10] provide additional details on procedures to attach test insects to flight mill arms, flight mill manufacture and calibration, data recording, and data file management.

Eight flight mills were set up in the same IQF room in which weevils were maintained. For each flight mill trial, eight weevils were randomly assigned to flight mills and weevils were tethered to flight mills in the morning, with set up occurring between 08:00 and 09:30. Prior to the commencement of experiments, the weight of each weevil was recorded on a digital balance (GF600, A & D Instruments, Elk Grove, IL, USA). Weevils were weighed again at the completion of experiments to determine weight change.

### 2.3. Statistical Analyses of Flight Distances and Velocities, Flight Bout Distances and Durations, Weight Loss, and Survivorship Times 

Prior to statistical analyses, data were checked for normality and, if necessary, research variables were subjected to Box–Cox procedures to determine power transformations to satisfy model assumptions (PROC TRANSREG [21]). The following transformations (where y = research variable) were made (if not indicated, the variable was not transformed before analysis): weight before trial; total distance flown: y^0.25^; velocity; maximum bout distance flown: y^0.25^; and maximum bout length: y^0.25^.

The weight of *R. palmarum* adults by gender and mating status (i.e., unknown vs. unmated) before trials was analyzed using a two-way analysis of variance (PROC GLM [21]). The procedure GENMOD in SAS [21] with binomial distribution and logit link function [22] was used to test if percentage weight loss over the course of experiments was influenced by gender, mating status (i.e., unknown vs. unmated), the 24 h flight trial (i.e., trials 1–14 only), and their interactions. No weevils flew >1 km for trials 15–17, and these were excluded from analyses. The response variable was the total percentage weight loss recorded per weevil per flight trial. Separate models were run for each of the two response variables: (a) % weight change between successive flights, and (b) % weight loss across successive flights as a function of initial starting weight. Tukey tests at the 0.05 level of significance were conducted to separate means when significant effects were detected.

For all other flight parameters of interest by gender, mating status, and flight trial, a linear mixed effects model for repeated measures data was used to make comparisons (PROC MIXED [21]). Fixed effects in the model included gender, mating status (i.e., unmated vs. unknown), trial number (i.e., one through fourteen only), and all the two-way interactions between variables. Repeated measurements were recordings of flight parameters recorded per each weevil for each flight trial. Separate models were conducted for each of the six flight variables: (1) total distance flown, (2) velocity, (3) mean maximum bout distance flown, and (4) mean maximum bout time length. Effective degrees of freedom for fit models were estimated using the Kenward–Rogers method (option ddfm = kr [21]) [23]. Pairwise comparisons for significant main effects were adjusted using the Tukey–Kramer method. Significance for all tests was set at *α* < 0.05.

Kaplan–Meier analyses were performed using PROC LIFETEST [21] on survival data for weevils. Kaplan–Meier curves, as a function of survival probability and days survived and survival probability and distance flown by adult weevils, were generated for each gender by mating status. These curves were subjected to a log-rank test in PROC LIFETEST [21] at the 0.05 level of significance to determine if significant differences in distances flown or days survived existed between unknown mating status and unmated male and female weevils.

### 2.4. Quantification of Dispersal and Redistribution Kernels for R. palmarum Using Distance Flown Data

No significant differences were detected as a function of gender or mating status when Kaplan–Meier analyses were completed (see Results). Consequently, all flight data by mating status and sex were combined and used to define dispersal curves and corresponding redistribution kernels.

Flight data were only used for analyses if experimental weevils flew >1 km, and flight data from the first 14 trials satisfied this requirement and were analyzed. Weevils tethered to flight mills for trials 15, 16, and 17 failed to fly >1 km and these flight trials were not included in analyses. Flight distance data for individual flights 1 (n = 82 weevils flew >1 km), 2 (n = 89), 3 (n = 80), 4 (n = 73), 5 (n = 65), 6 (n = 52), 7 (n = 40), 8 (n = 26), 9 (n = 21), and combined flights, flights 10–14 (Flight 10 [n = 8], 11 [n = 8], 12 [n = 6], 13 [n = 2], 14 [n = 1]), as well as all flights combined (1–14), were divided across distance flown bins according to Sturges’ formula, where the number of distance bins used per flight trial = 1  +  log2 (n) (n = number of observations [24]) and maximum flight distance per trial was used as the upper bin limit. Binned flight (number of bins used ranged 5–7) data were used to generate a frequency histogram for each flight trial and all flights combined and the mid-point in each bin was identified. To these midpoints, five different dispersal curves (see Kot et al. [1] for equations for model curves 1, 2, 3, 4, and 7) with finite integrals were examined for goodness of fit to binned data using sums of squares error (SSE) and coefficient of determination (R^2^ = 1 − SSE/total sums of squares (SST)). Curve equation parameters were determined using PROC NLIN [21] and the best (determined by size of SSE (nonlinear model with the smallest value of residual sum of squares (RSS) indicated the best fit to the data)) parameterized equation standardized by multiplying by bin width, number of weevils flown per flight trial, and a normalizing constant specific to each individual flight trial and combined flight trials (see Kot et al. [1] for normalizing constant calculations) were fitted to binned flight data specific to each analysis for each flight trial. Dispersal curve equations were parameterized and normalized to provide an area under the curve of 1 when reflected about the origin, which generated the redistribution kernel for the distances flown by weevils for each individual flight trial and trial combinations [1]. The best fitted models to individual and combined flight data sets were tested for kurtosis using the following equation:

$$\mathrm{Excess}\;\mathrm{Kurtosis}\;(k)\;=\;\frac{\int x^{4}f\left(x\right)dx}{{\left[\int x^{2}f\left(x\right)dx\right]}^{2}}-3$$

which was solved using the option vardef = n in PROC MEANS [21]. Values of *k* > 0, *k* = 0, and *k* < 0 indicate leptokurtosis, mesokurtosis, and platykurtosis, respectively.

## 3. Results

### 3.1. Flight Distances and Velocities, Flight Bout Distances and Durations, Weight Loss, and Survivorship Times

#### 3.1.1. Flight Distances and Velocities 

Mean total distances flown and mean flight velocity for each flight trial did not differ by sex, mating status, sex by mating status, sex by flight trial, or mating status by flight trial (Table 1). 

**Figure 1:**
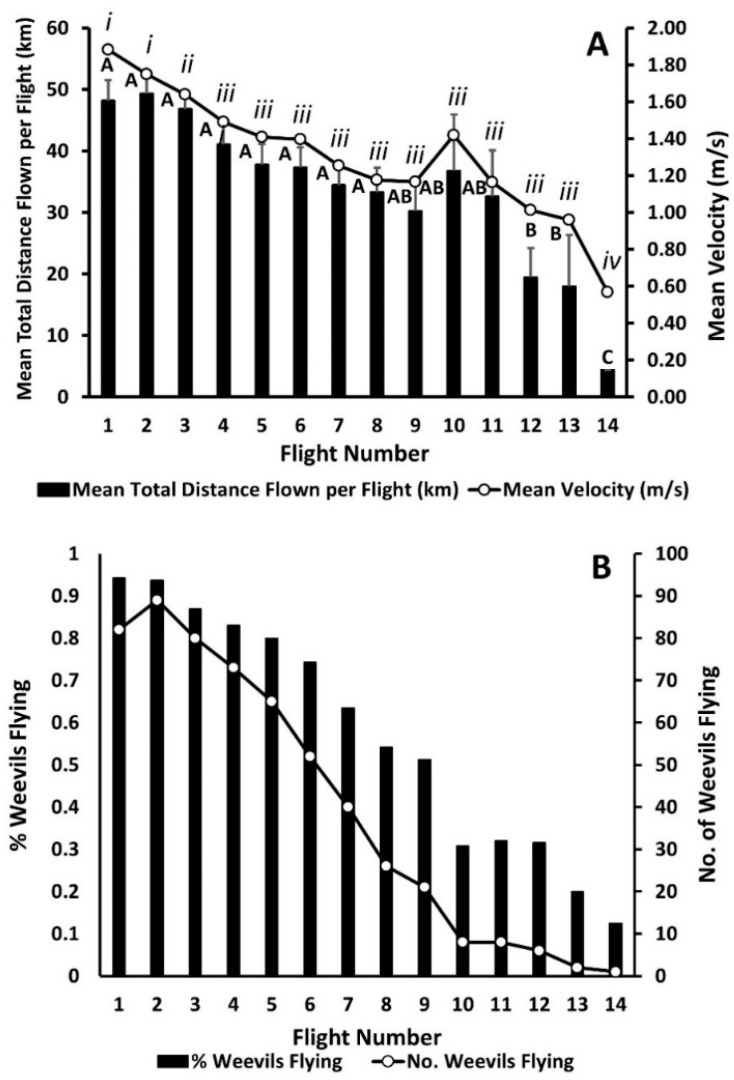
(**A**) Mean (+SE) total distance flown (km) per weevil flight trial and mean flight velocity (m/s) per weevil per flight trial across 14 consecutive flight trials. Data points with different letters or Roman numerals indicate significant differences at the 0.05 level for mean total distance flown and mean velocity, respectively. (**B**) Percentage and number of weevils flying >1 km per flight trial.

Significant differences in average distance flown and average flight velocity across consecutive flights were observed (Table 1; Figure 1A). The trend in mean distances flown began to decrease after flight two and mean flight velocity showed a decreasing trend beginning after the first flight, and a marked reduction in flight capabilities was observed after trial 12 (Figure 1A). The number and corresponding percentage of weevils flying per trial likewise declined across successive flights (Figure 1B). The average cumulative life time distance flown by female weevils that were unmated or of unknown mating status was 219.55 ± 21.14 km (range: 10.40–806.29 km; median cumulative distance flown was 207.62 km). The average cumulative life time distance flown by male weevils that were unmated or of unknown mating status was 267.72 ± 33.24 km (range: 16.36–758.49 km; median cumulative distance flown was 204.91 km).

#### 3.1.2. Flight Bout Distances and Durations 

**Figure 2:**
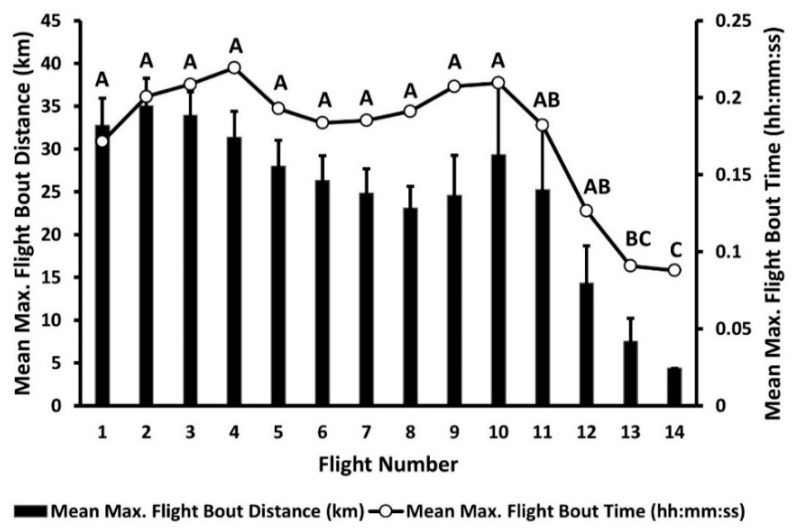
Mean (+SE) maximum flight bout distance (km) and mean maximum flight bout time (hh:mm:ss) exhibited by adult *Rhynchophorus palmarum* across 14 consecutive flight trials. Data points with different letters indicate significant differences at the 0.05 level for both data sets.

Mean maximum flight bout distance, mean maximum flight bout time, mean flight bout distance, and mean flight bout time for each flight trial did not differ by sex, mating status, sex by mating status, sex by flight trial, or mating status by flight trial (Table 1). Average maximum flight bout distances and their associated average times of duration differed significantly across trials, with significant declines in duration and time being observed after flight trial 10 (Table 1; Figure 2). The longest individual maximum flight bout recorded was 154.54 km on the first flight by a female of unknown mating status, which flew uninterrupted for 17 h 20 min and 39 s with an average flight velocity of 2.25 m/s and a maximum recorded velocity of 4.00 m/s. The second longest individual flight bout recorded was 150.85 km by a male weevil of unknown mating status for flight 4, which flew constantly for 20 h 41 min and 35 s with an average velocity of 2.01 m/s and a maximum recorded velocity of 3.41 m/s.

#### 3.1.3. Weevil Weight Loss 

**Figure 3:**
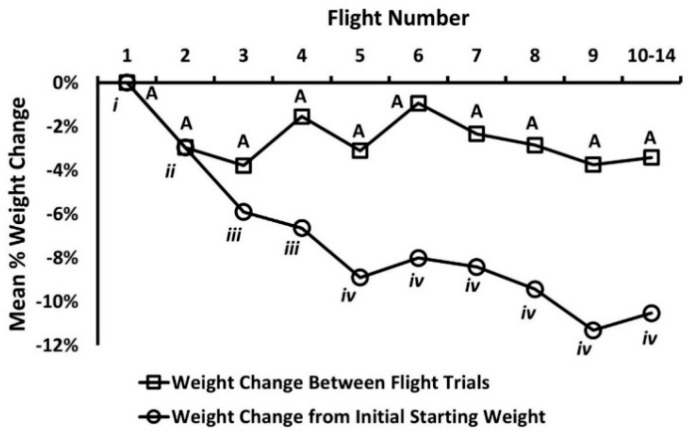
Mean percentage change in weight for adult *Rhynchophorus palmarum* across successive flight trials and in relation to initial starting weight at preceding flight or as function of weight at the commencement of flight trial one. Data points with different letters indicate significant differences at the 0.05 level for weight change from weight at preceding flight trial (upper case letters) or initial starting weight at commencement of flight 1 (Roman numerals).

No significant differences in pre-trial weights were detected for experimental weevils used in flight trials based on sex (*F* = 0.53; df = 1, 97; *p* = 0.47), mating status (*F* = 0.15, df = 1, 97; *p* = 0.70), and their interaction (*F* = 0.09; df = 1, 97; *p* = 0.76). Mean percentage weight loss of adult weevils across successive flights as a function of weight in the preceding flight did not differ by sex (*χ*^2^ = 1.07, df = 1, *p* = 0.31), mating status (*χ*^2^ = 0.24, df = 1, *p* = 0.62), flight trial (*χ*^2^ = 14.73, df = 9, *p* = 0.98), sex by mating status (*χ*^2^ = 0.05, df = 1, *p* = 0.97), sex by flight trial (*χ*^2^ = 1.71, df = 9, *p* = 0.09), or mating status by flight trial (*χ*^2^ = 14.96, df = 9, *p* = 0.09) (Figure 3). In comparison, a significant reduction in average percentage weight loss was observed for weevils when weights were compared with initial starting weights at the commencement of flight trial 1 (*χ*^2^ = 43.51, df = 9, *p* < 0.0001; Figure 3). By the end of flight trials that provided useful flight data, surviving weevils had lost, on average, ~11% of their body weight when compared with initial weights at flight 1 (Figure 3).

#### 3.1.4. Weevil Survivorship Times and Flight Activity by Age 

**Figure 4:**
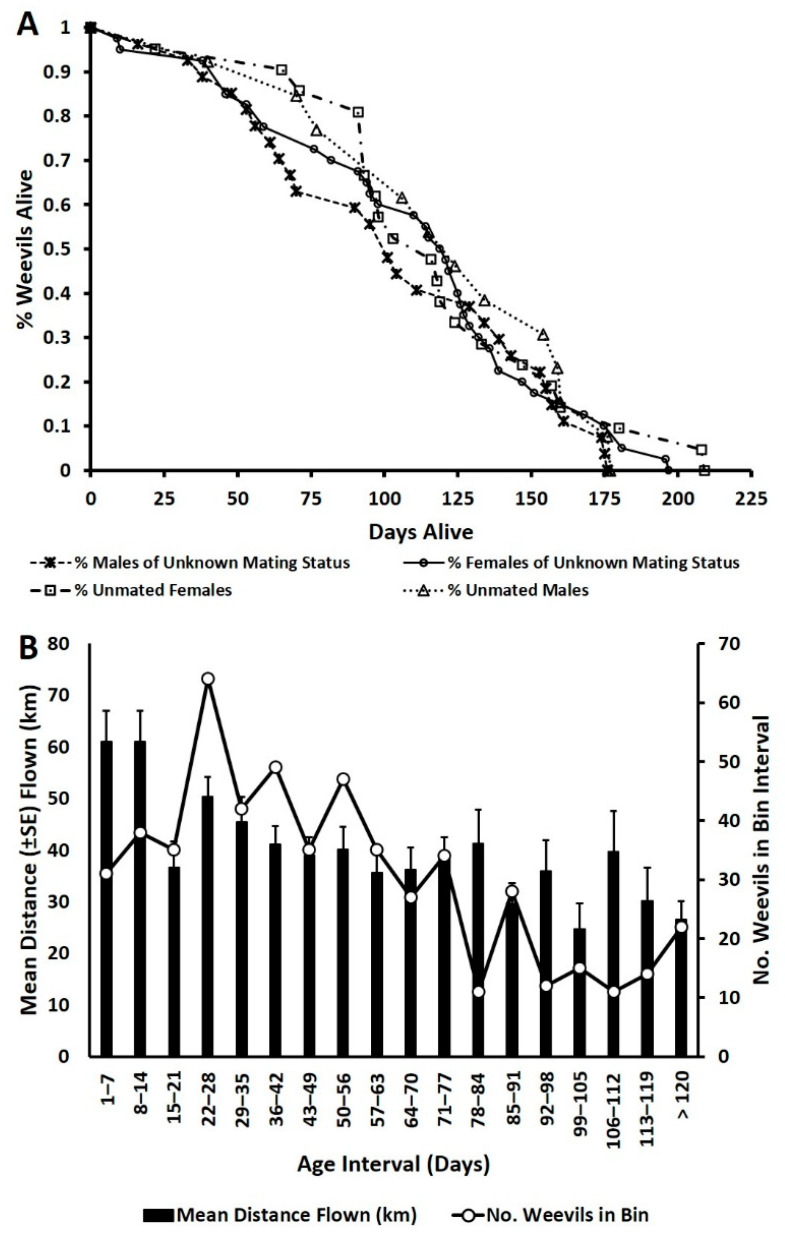
(**A**) Survivorship curves for adult *Rhynchophorus palmarum* used for flight mill trials. (**B**) Mean (+SE) distance flown (km) and number of weevils that flew >1 km in age interval bins.

The log-rank test assessing the probability of days survived by adult weevils was not significant for gender by mating status (*χ*^2^ = 0.92, df = 3, *p* = 0.82), mating status (*χ*^2^ = 0.33, df = 1, *p* = 0.57), or sex (*χ*^2^ = 0.41, df = 1, *p* = 0.52) (Figure 4A). Adult male weevils of unknown mating status lived for an average of 98.15 ± 9.45 days (range 12–176 days; median age 101 days). Female weevils of unknown mating status lived for an average of 106.18 ± 7.64 days (range 9–196 days; median 117 days). Unmated females lived for an average of 113.00 ± 9.10 days (range 22–208 days; median 103 days). Unmated males lived for an average of 112.92 ± 12.27 days (range 40–176 days; median 115 days). Weevils that flew >1 km in each age interval exhibited strong capacity for flight as they aged (Figure 4B). Of the 22 weevils that flew >1 km in age category >120 days, the range of flight distances was 2.05–77.61 km (mean distance flown = 26.56 ± 3.61 km; median 25 km) (Figure 4B).

### 3.2. Dispersal and Redistribution Kernels for R. palmarum Using Distance Flown Data

The log-rank test assessing the probability of days survived by adult weevils was not significant for gender by mating status, mating status, or sex (see above). Similarly, the log-rank test assessing the probability of distance flown by adult weevils was not significant for gender by mating status (*χ*^2^ = 3.78, df = 3, *p* = 0.29), mating status (*χ*^2^ = 0.01, df = 1, *p* = 0.93), or sex (*χ*^2^ = 2.21, df = 1, *p* = 0.14). Therefore, all flight data by mating status and sex were combined and the best fitting of the five curves analyzed from Kot et al. (1) was determined to be curve 1 for flights 1, 4, 6, 7, 9, and 1–14 and curve 3 for flights 2, 3, 5, 8, and 10–14 (Table 2).

**Figure 5:**
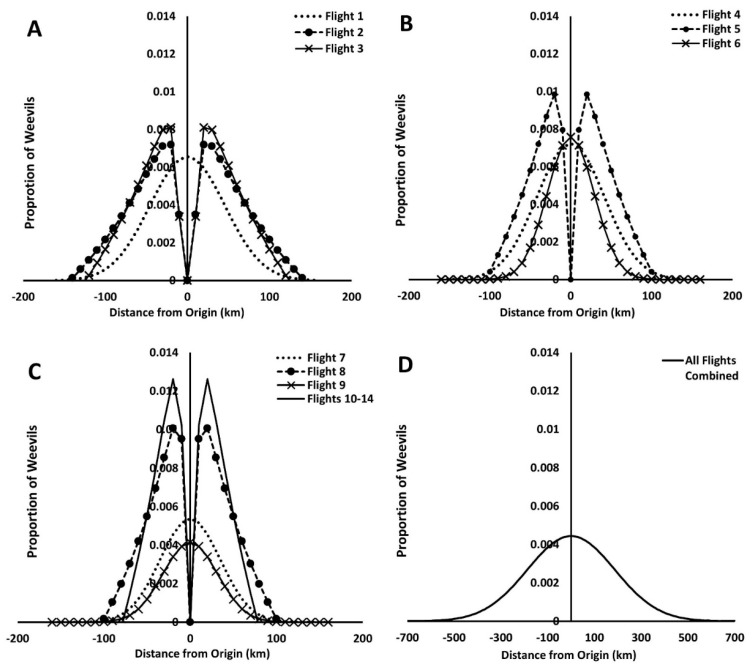
Redistribution kernel graphs for (**A**) flights 1–3, (**B**) flights 4–6, (**C**) flights 7–9 and 10–14 combined, and (**D**) all flights (1–14) combined. Graph equations were parameterized and normalized and using estimates from Table 2. All curves exhibit platykurtosis.

These two functions were used to generate the corresponding redistribution kernel for each flight (Figure 5). The excess kurtosis measures, k, were all <0, indicating that the equations describing the curves that were produced were all platykurtic (Table 2) (Figure 5). Redistribution kernel plots for flights 2, 3, 5, 8, and 10–14 combined produced graphs with bimodal peaks centered at the origin (Figure 5).

## 4. Discussion

This study is one of the first to investigate the capacity of study insects to undergo repeat flights on flight mills over the course of their natural lifespan. Flight mill studies under controlled laboratory conditions indicate that adult *R. palmarum* are strong fliers and male and female weevils, regardless of sex or mating status, are capable of multiple long distance flights over their lifetimes. Repeat flight mill assays by Barkan et al. [20] with *R. ferrugineus* demonstrated that this invasive palm weevil was capable of multiple repeat flights that averaged ~62 km for adult weevils. In comparison, male and female *R. palmarum* averaged ~268 km and ~220 km, respectively. In this study, the maximum cumulative flight distance of ~806 km (average flight distance across nine consecutive flights that were >1 km was ~90 km with a maximum flight distance of ~151 km recorded for flight 5) was attained by an unmated female. In contrast, the maximum cumulative life time flight distance for an individual male *R. ferrugineus* that was permitted to fly for 3 h per trial (vs. 24 h for the work reported here) across 11 flight trials was ~315 km (average flight distance was ~29 km) [20]. For *R. palmarum*, average cumulative flight distances and associated velocities and flight bout distances and durations declined across successive trials. This outcome may have resulted from increasing age, accumulating physiological stress, inadequate diet, or a combination of these factors as the number of repeat flights increased. An important caveat for interpreting these flight distance data stems from the fact that they are laboratory generated and only provide an indication of potential flight capabilities should *R. palmarum* choose to initiate multiple consecutive flights in nature. It is unknown if weevils engage in such flight activity in the field and behavioral factors that initiate flight in *R. palmarum* are also poorly understood. As Kissling et al. [5] note, tracking flying insects over long distances in the field is extremely difficult and tools to do this easily are not currently available.

The redistribution kernels generated from flight distance data for *R. palmarum* across 14 trials were all platykurtic and exhibited negative excess kurtosis, indicating that the tails of these curves have fewer extreme or outlier observations (i.e., they are light tailed) than would be expected for data with a normal distribution. The peak morphology of redistribution graphs was either unimodal or bimodal around the origin. However, kurtosis is not characterized by peak morphology (i.e., peakedness) of distribution data and data kurtosis is defined solely by the tails (i.e., tailedness) of the probability distribution [25,26].

The consistency of *R. palmarum* flight data exhibiting only platykurtic distributions across all flight trials was unexpected. It is uncertain as to whether this observation was a chance artifact from repeatedly using the same cohort of weevils for experiments, whether consistency of dispersal distributions is characteristic of pest populations at the leading edges of invasion events, or if it is a signature-like species-specific phenomenon. These possibilities could be tested by repeating flight mill trials with *R. palmarum* sourced from a centralized region of the native range (e.g., Costa Rica, Colombia, or Brazil). A comparison of very similarly generated flight mill data for single 24 h flights for *R. ferrugineus*, *R. vulneratus*, and *R. palmarum* indicated that each species had unique dispersal distributions that were mesokurtic, leptokurtic, and platykurtic, respectively [4]. When these distribution data were plotted together, the tails of the platykurtically (i.e., “thin” tailed) distributed flight data for *R. palmarum* were “fatter” than the leptokurtically (i.e., “fat” tailed) distributed data for *R. vulneratus*. The platykurtic redistribution kernel graph for *R. palmarum* had a greater proportion of weevils sitting in the curve tails at distances further from the origin than the leptokurtic redistribution kernel graph generated for *R. vulneratus* [4]. This contrast suggests that the magnitude of what constitutes “thin” and “fat” tails with respect to kurtoses of dispersal data needs careful consideration when assessments of potential invasion risk and rates of spread are being made using data generated across different studies.

*Rhynchophorus palmarum* exhibited steady weight loss over the course of repeat flight assays even though adult weevils readily consumed apples, bananas, and sugar cane in captivity. On this diet, experimental male and female weevils, irrespective of mating status, lived for an average of ~108 days. When percentage weight loss was assessed as a function of initial weight at the time of flight 1 (i.e., commencement of repeat flight trials), weevils exhibited a steady irreversible decline in weight, with an average weight loss of ~11% being observed for combined trials 10–14, the final set of flight assays that provided useable flight data. However, despite feeding and rest periods, full weight recovery to the initial starting weight across flight assays was not observed. Consistent weight loss as a function of initial starting weight at time flight assays commenced was likely due to energy expenditure and physiological deterioration from repeat flight trials, aging, and possibly a substandard diet.

Despite demonstrating an ability to undertake numerous long distance flights on flight mills, the spread of *R. palmarum* throughout San Diego County has not been rapid. Since first detection in Tijuana, Mexico in 2010, *R. palmarum* had moved northwards and established populations as far north as San Marcos in San Diego by 2020, a distance of ~82 km. This suggests movement at the leading edge of the invasion may only be ~8 km/yr. This observation may be indicative that natural dispersal conforms to a thin-tailed platykurtic distribution, as suggested by flight mill data generated in this study. Alternatively, urban, recreational, and commercial areas in San Diego County have abundant plantings of palms, of which the most common is *P. canariensis*, a highly preferred host for *R. palmarum*.

Given this palm-rich environment, there may be relatively little impetus for weevils to fly long distances as new hosts are plentiful and in close proximity to each other. Additionally, factors that motivate innate dispersal behaviors, including repeat flights, are unknown, and it is uncertain if weevils undertake additional flights after colonizing suitable palm hosts. However, should weevils find themselves in areas lacking hosts (e.g., wilderness areas characterized by chaparral or desert), they may have the capacity to clear these inhospitable zones and colonize new regions. These types of long distance dispersal events, should they occur, may unexpectedly accelerate spread into new areas.

An important agricultural area vulnerable to incursion is the edible date groves (i.e., *P. dactylifera*, a known host for *R. palmarum*) in the Coachella Valley, a desert area that is a linear distance of ~150 km across inhospitable and host-poor terrain from current *R. palmarum infestations* in San Diego County. The edible date industry in California is valued at $100 million per year [27]. The possibility of long distance spread that threatens agricultural enterprises emphasizes the need for a well-coordinated and systematic detection and monitoring program, which is currently lacking for *R. palmarum* in California even though trap optimization studies have been completed [28,29]. Flight distance data and distribution of these data obtained from repeat flight mill assays presented here can aid in the development of coordinated area-wide monitoring and management programs in California for *R. palmarum*.

## Figures and Tables

**Table 1:** Results of repeated measures analyses examining the effects of gender, mating status (i.e., unmated vs. unknown), the 24 h flight trial (i.e., one through fourteen), and their interactions on (**A**) total distance flown, (**B**) velocity, (**C**) mean maximum bout distance flown, and (**D**) mean maximum bout time length recorded across experimental weevils tethered to flight mills over their life time (i.e., flights one through fourteen).

(A) Distance Flown	Num *df*	Den *df*	*F*	*p*
Gender (G)	1	147	2.28	0.133
Mating status (M)	1	167	0.28	0.599
Flight (F)	13	431	6.48	<0.0001 *
G × M	1	83.2	3.31	0.072
G × F	11	431	1.46	0.063
M × F	11	430	1.16	0.498
**(B) Velocity**	**Num *df***	**Den *df***	***F***	***p***
Gender (G)	1	152	0.03	0.974
Mating status (M)	1	179	1.75	0.188
Flight (F)	13	437	10.24	<0.0001 *
G × M	1	83.9	3.14	0.511
G × F	11	437	1.08	0.379
M × F	11	436	1.62	0.089
**(C) Maximum Bout Distance Flown**	**Num *df***	**Den *df***	***F***	***p***
Gender (G)	1	159	0.72	0.398
Mating status (M)	1	182	0.87	0.352
Flight (F)	13	436	3.04	0.001 *
G × M	1	85.1	3.58	0.062
G × F	11	436	1.58	0.073
M × F	11	435	1.45	0.148
**(D) Maximum Bout Length**	**Num *df***	**Den *df***	***F***	***p***
Gender (G)	1	188	0.08	0.774
Mating status (M)	1	170	0.27	0.601
Flight (F)	13	434	2.16	0.039 *
G × M	1	88.4	1.59	0.211
G × F	11	435	1.56	0.101
M × F	11	433	0.99	0.451

* Indicates significance at the 0.05 level.

**Table 2:** Lowest calculated RSS values fitted to equations for curves 1 and 3 from Kot et al. [1], parameter estimates and normalizing constants for curves 1 and 3, and measures of excess kurtosis for individual flights 1–9, combined flights 10–14, and all flights combined (1–14).

	Flight Number										
RSS values	1	2	3	4	5	6	7	8	9	10–14	All flights
Curve 1	50.01	-	-	94.71	-	10.49	6.77	-	5.59	27.69	30.77
Curve 3	-	65.63	84.73	-	49.24	-	-	2.91	-	-	-
	**Curve Parameter Estimates**										
*t*	-	7.63	8.07	-	5.68	-	-	4.64	-	5.9	-
*s*	-	143.09	124.07	-	104.89	-	-	101.91	-	78.32	-
*a*	3.30	164.2	160.3	3.23	95.15	2.53	2.56	30.33	2.12	63.84	3.43
*b*	0.00014	32.02	31.99	0.000164	19.74	0.00018	0.00009	6.36	0.00005	13.96	0.00000062
*c*	-	−757.1	−754.8	-	−345.8	-	-	−95.40	-	−232.9	-
Normalizing constant	4149.03	4226.40	3299.92	3500.40	1901.42	1668.01	2406.76	647.68	1999.74	822.5	69497.49
	**Excess Kurtosis Estimates**										
*k*	−1.11	−1.22	−1.25	−0.92	−1.05	−0.87	−1.12	−1.03	−1.41	−1.60	−0.86

## Data Availability

The data presented in this study are available on request from the corresponding author. The data are not publicly available due to privacy.
